# Population-based colorectal cancer screening programmes using a faecal immunochemical test: should faecal haemoglobin cut-offs differ by age and sex?

**DOI:** 10.1186/s12885-017-3555-3

**Published:** 2017-08-29

**Authors:** Eunate Arana-Arri, Isabel Idigoras, Begoña Uranga, Raquel Pérez, Ana Irurzun, Iñaki Gutiérrez-Ibarluzea, Callum G. Fraser, Isabel Portillo, EUSKOLON Group, José Luis Hurtado, Carmen de No, Carlos Enciso, Maite Escalante, Begoña Atarés, José Javier Aguirre, Esther Pereda, Edurne Marañón, Pedro Otazua, María Fernández, José Francisco Egido, Eva Zapata, Leire Zubiaurre, Juana Mari Rodríguez, Pedro Esteban Sampedro, Marisa Goyeneche, José María Arrinda, Mari Luz Jauregui, Marta Gómez, Marta Saiz, Luis Bujanda, Inés Gil, Isabel Montalvo, José Miguel Larzabal, Maddi Garmendia, Fernando Izquierdo, Francisco Javier Fernández, Iago Rodríguez, Alain Huerta, Eduardo de Miguel, Inmaculada Barredo, Fidencio Bao, Anaiansi Hernández, Isabel Rodriguez, María José Fernández-Landa, María Imaz, Angel Calderón, Francisco Polo, Nagore Arbide, Gaspar Lantarón, Cristina Quesada, Itziar Marzana, Enrique Ojembarrena, Haritz Cortés, Iñaki Casado, Manuel Zaballa, Mar Ramírez, Amaia Aperribay, Cristian Amezaga, Lorea Martínez-Indart, Iraide Indart, Ariane Imaz-Ayo, Natale Imaz-Ayo, María José Fernández-Landa, Marta de la Cruz, Joseba Bidaurrazaga, Nerea Muniozguren, Nerea Larrañaga, Covadonga Audicana, Isabel Bilbao, José Luis Bilbao, Eduardo Millán, Saloa Unanue, Nere Mendizábal, Carlos Saiz, Santiago Rodríguez

**Affiliations:** 1 BioCruces Health Research Institute, Plaza Cruces 12, 48903 Barakaldo, Bizkaia Spain; 2 Colorectal Cancer Screening Programme Coordination Center, Bilbao, Spain; 3 Clinical Biochemistry Service, Donostia University Hospital, Basque Health Service, Donostia, Gipuzkoa Spain; 4 Clinical Biochemistry Service, Cruces University Hospital, Basque Health Service, Barakaldo, Bizkaia Spain; 5 Osteba, Basque Office for Health Technology Assessment, Ministry for Health, Vitoria-Gasteiz, Spain; 6 Centre for Research into Cancer Prevention & Screening, University of Dundee, Dundee, Scotland

**Keywords:** Adenoma, Colorectal cancer, Faecal immunochemical test, Faecal occult blood test, Interval cancers, Screening

## Abstract

**Background:**

The Basque Colorectal Cancer Screening Programme has both high participation rate and high compliance rate of colonoscopy after a positive faecal occult blood test (FIT). Although, colorectal cancer (CRC) screening with biannual (FIT) has shown to reduce CRC mortality, the ultimate effectiveness of the screening programmes depends on the accuracy of FIT and post-FIT colonoscopy, and thus, harms related to false results might not be underestimated. Current CRC screening programmes use a single faecal haemoglobin concentration (f-Hb) cut-off for colonoscopy referral for both sexes and all ages. We aimed to determine optimum f-Hb cut-offs by sex and age without compromising neoplasia detection and interval cancer proportion.

**Methods:**

Prospective cohort study using a single-sample faecal immunochemical test (FIT) on 444,582 invited average-risk subjects aged 50–69 years. A result was considered positive at ≥20 μg Hb/g faeces. Outcome measures were analysed by sex and age for a wide range of f-Hb cut-offs.

**Results:**

We analysed 17,387 positive participants in the programme who underwent colonoscopy. Participation rate was 66.5%. Men had a positivity rate for f-Hb of 8.3% and women 4.8% (*p* < 0.0001). The detection rate for advanced neoplasia (cancer plus advanced adenoma) was 44.0‰ for men and 15.9‰ for women (*p* < 0.0001). The number of colonoscopies required decreased in both sexes and all age groups through increasing the f-Hb cut-off. However, the loss in CRC detection increased by up to 28.1% in men and 22.9% in women. CRC missed were generally at early stages (Stage I-II: from 70.2% in men to 66.3% in women).

**Conclusions:**

This study provides detailed outcomes in men and women of different ages at a range of f-Hb cut-offs. We found differences in positivity rates, neoplasia detection rate, number needed to screen, and interval cancers in men and women and in younger and older groups. However, there are factors other than sex and age to consider when consideration is given to setting the f-Hb cut-off.

## Background

Colorectal cancer (CRC) screening using tests for the presence of blood in faeces, commonly known as faecal occult blood tests (FOBT), has been shown to be an effective intervention for reducing CRC-related mortality in controlled studies conducted both in Europe [1–3] and in the USA [4]. The mortality reduction varied between 14 and 18%, with colonoscopy being used as the second stage investigation in those with a positive faecal test result. Thus, screening reduces the burden of CRC, which is the most common cancer in industrialized countries and has a high mortality rate of approximately 25.4 expected deaths per 100,000 in the overall population. The standardized incidence-based mortality ratio is 0.47 (95% confidence interval [CI]: 0.26–0.80) with colonoscopic polypectomy, suggesting a 53% reduction in mortality [5, 6].

FOBT has been widely implemented for CRC screening and, in 2003, the European Union (EU) published an official recommendation for its members to carry out FOBT screening for the average-risk population aged between 50 and 74 years [7]. In this regard, faecal testing has improved markedly since the aforementioned studies were carried out, with the original guaiac test (gFOBT) being superseded by faecal immunochemical tests for haemoglobin (FIT), which are potentially much better at detecting advanced adenomas (AA) and CRC and are also much better accepted by potential participants because of ease of use and the lack of a need for special dietary requirements [8, 9]. The EU guidelines recommend use of FIT in population-based programmes [10, 11] and, indeed, an impact on cancer incidence has been found in recent studies [12, 13], although further investigation is needed to assess the longer-term impact. A recent meta-analysis shows an average sensitivity of 79% and a specificity of 94% of FIT for CRC in asymptomatic subjects [14].

Current main concerns are centered on quality-assurance practices and the possible negative consequences of such programmes. Quality assurance throughout the screening process is based on criteria and indicators recommended by the European guidelines [10], whereas the negative effects concern the main side effects of CRC programmes, in particular, colonoscopy-related complications and false-negative and false-positive results. In the case of false positive results, three studies found differences between the sexes [15, 16] and noted that this situation was unsatisfactory, especially for women [17].

Some models have been designed to include faecal haemoglobin concentration (f-Hb) as a predictor for colorectal neoplasia and have suggested that adjustments must be made to take into account sex, family history or morbidities when implementing programmes [18], In this regard, the Scottish Bowel Screening Programme evaluation using FIT showed important differences in the results for men and women, with a greater participation with FIT than with gFOBT, a higher positivity rate in men than women in all groups, and a higher detection rate in men for AN and CRC. In contrast, the number of false-positive results was lower in men (49.1% versus 58.9% in women) for colonoscopies performed [19]. A similar pattern was reported by the Basque Country for lesions detected in the period 2009–2011 [20].

Adjusted incidence rates for CRC in the Basque Country have increased significantly, by 2.3% per year in men (from 60.3 per 100,000 in 2000 to 87.6 in 2011) and by 6.5% per year in women (from 56.6 in 2007 to 71.8 in 2011). The age-standardized incidence rates for 2007 (prior to implementation of the Basque Country Colorectal Cancer Screening Programme) showed a high men-to-women ratio for different locations [21].

A recent review [22] concluded that the influence of sex on the comparative performance of tests for detecting advanced colorectal neoplasia (AN) has not been investigated with sufficient power in any of the diagnostic cohort studies conducted to date. In a prospective cross-sectional study, van Turenhout et al. [23] concluded that FIT has a higher sensitivity and lower specificity for CRC in men and that different f-Hb cut-offs should be used in screening programmes. These data are consistent with those published by Fraser et al. [24], who concluded that f-Hb distributions vary by sex and age, this supporting the view that setting and using a single f-Hb cut-off in any CRC screening programme is far from ideal. Alvarez-Urturi et al. [25] have recently conclude in the ColonPrev randomized controlled trial study that FIT cut-offs could be individualized by sex and age to improve the performance of FIT in CRC screening programmes. On the other hand Kapidzic et al. [26], in a prospective cohort of invited people from the Dutch population-based screening programme, do not recommend different f-Hb cut-offs in men and women based on the consideration that positive predictive values for the sexes should be the same. Establishing different f-Hb cut-offs between men and women and between age groups could influence the effectiveness of screening. Looking ahead to achieve consistent detection rates among regions, the cut-offs could differ. However any increase in the f-Hb cut-off selected to define positivity, while increasing sensitivity for AN, can increase the rate of false positives [27].

Colonoscopy demand increases with the use of FIT when used with the widely applied low f-Hb cut-offs since the expected number of positive test results is more than three times higher than that with gFOBT, posing an economic challenge for many regions as regards the implementation of population-based screening programmes, since additional investment and resources are needed to implement them, at least in the early screening rounds. As such, an exercise to estimate the clinical outcomes including the number needed to screen (NNS) to detect one case, and the f-Hb cut-offs to be used are a difficult dilemma for epidemiologists and decision-makers. Using quantitative FIT, the f-Hb cut-off (s) to be used becomes a crucial decision since the positivity rate determines the number of colonoscopies required. In this regard, some f-Hb cut-offs have been suggested and simulated outcomes created to answer these questions [28–30].

The main question, however, is how to determine the best f-Hb cut-off (s) for a specific target population in order to detect the true positive results without increasing the number of interval cancers (ICs), a serious consideration in any screening programme [31, 32]. In this study, we aimed to answer these questions on the basis of a high participation rate population-based screening programme and determine whether strategies using f-Hb cut-offs stratified by sex and age group may be useful.

## Methods

### Study population and interventions

The Basque Country CRC Screening Programme is population-based and started in 2009 as a pilot and was extended in 2010 after evaluation and optimisation of the processes involved. The main strategy was based on: A) a Coordinating Office, including clinical epidemiologists and statisticians, to plan, organize and manage the programme; B) all residents from 50 to 69 years were invited, taking into account the Health Centers and referral Hospitals, in order to adjust the positivity expected and colonoscopy capacity; C) prior to the invitation, the Coordinating Office selected the target population and linked the database to the Basque Population Cancer and Medical Procedures Registries to exclude people with a previously diagnosed CRC, terminal illness and colonoscopy reported in the last 5 years; D) training and involvement of Basque Health Service Primary Care staff; E) individualized posted invitations providing information about the programme. After 4–6 weeks from the initial invitation, the kit was sent along with instructions and an individualized bar code. This code allows the sample and person to be identified when processing the result. Samples were collected at Primary Health Centers of the Basque Public Health Service and processed in centralized public laboratories under strict total quality management systems; F) automatically the software system introduces the result in the “ad hoc” CRC database and primary care physicians review all results of their patients (reader has to bear in mind that electronic clinical records are implemented in community care in the Basque Country). Letters were posted with the results: a) if negative, the invitation will be repeated in 2 years’ time if the person is younger than 70 years, or b) if positive, participants are recommended to visit their General Practitioner, who will indicate the need for a colonoscopy and c) in case of error, another kit and instructions were sent; G) colonoscopies are performed in referral public hospitals under sedation by expert specialists; H) all cases are followed-up with close coordination between Primary Care and Specialized Units; J) every case is coded by the Coordinating Office staff following standard EU guidelines and Spanish Network consensus recommendations [10, 33]. This study was approved by the Basque Country’s Ethics Committee (Reference: PI2014059). All participants provide written informed consent.

Detection of ICs: prior to a subsequent invitation, all negative cases from a previous round are linked to the register of hospital discharges with ICD-9 1530–1548, in primary and secondary diagnosis, ICDO-10 C18-C21 of hospital registers and population-based Cancer registries as well as codes of Pathology. In all coinciding cases, the qualified staff from the Programme’s Coordinating Centre checked the clinical history, including the cases as ICs which complied with the criteria of having a negative FIT result in the previous invitation (0–24 mo or more in case of a delay in the invitation to the screening programme). To ensure against any possible losses, this process was repeated on an annual basis.

### Definitions

The FIT used from early 2009 and in early 2010 (during the pilot study) were OC-Sensor Micro (Eiken Chemical Co, Tokyo, Japan) and FOB-Gold (Sentinel CH. SpA, Milan, Italy), in both with a f-Hb cut-off of 20 μg Hb/g faeces. After comparison of the results obtained with both devices [34], OC-Sensor was selected and has been used since. OC-Sensor is a quantitative FIT, with chemistry based on human haemoglobin antibody mediated latex agglutination. Bar coded specimen collection devices were analysed for f-Hb. In the current analysis, the data are only related to this FIT. The result was considered positive when f-Hb was ≥20 μg Hb/g faeces.

The histology of all lesions detected was evaluated by expert pathologists specializing in gastrointestinal oncology according to the quality standards of the European guidelines [10]. The maximum reach of the endoscope, adequacy of bowel preparation, as well as the characteristics and location of any polyps were recorded. Adenomas ≥10 mm, adenoma with a villous component (i.e., tubulovillous or villous adenoma) or adenomas with severe/high-grade dysplasia were classified as AA [10].

AN was defined as CRC plus AA. Tumour staging was established according to the TNM classification system in agreement with the AJCC Cancer Staging Manual [35]. Finally, participants were classified and then assigned according to the most advanced lesion found.

### Statistical analysis

CRC screening performance measures were assessed following the European guidelines [10]. Variables were calculated and described as percentages with 95% confidence intervals.

The number needed to screen (NNS) was calculated as the number of completed screening tests required to find one AN. All test characteristics were calculated separately for f-Hb cut-offs of 20, 25, 30, 35, 40, 50 and 60 μg Hb/g faeces, respectively.

Differences in the test characteristics between men and women and different age ranges were assessed using the chi-squared and/or Fisher’s tests. Since the data on f-Hb did not follow a normal distribution, the Mann-Whitney U test was used to compare continuous variables between the groups. The normality of the distribution of continuous variables was assessed using a normal Q-Q plot. A *p*-value of less than 0.05 was considered to be statistically significant using a two-sided test.

A logistic regression was performed to analyze the risk of loss in the detection of AN by sex and age stratified group.

The statistical analysis was conducted using SPSS version 23.0 (IBM Corp. Released 2013. IBM SPSS Statistics for Windows, Version 23.0. Armonk, NY: IBM Corp.).

## Results

**Fig. 1:**
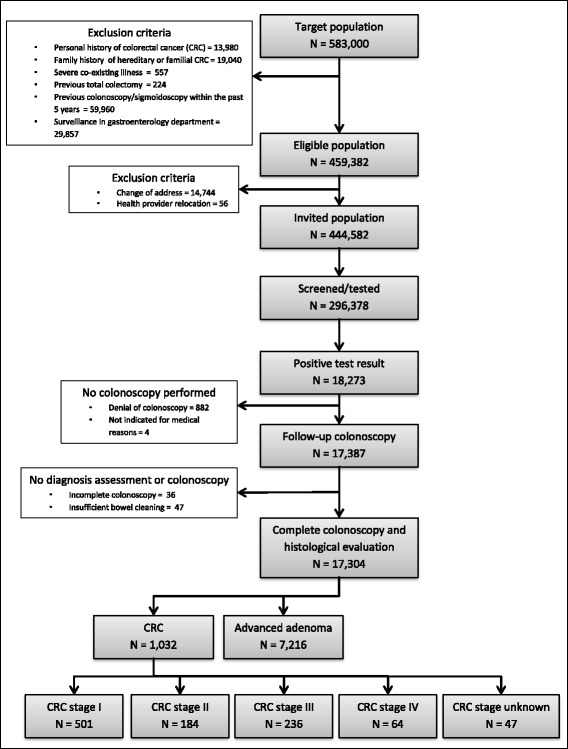
Study flow diagram

Between 2009 and 2012, 444,582 subjects were invited to the Basque Country CRC Screening Programme. The flow diagram is summarized in Fig. 1. The study population comprised 17,387 participants with a positive test result who underwent complete colonoscopy.

The overall participation was high (66.5%; 95% CI: 66.4–66.7), as was the colonoscopy compliance (95.1%; 95% CI: 94.8–95.5). The characteristics of the participants in the study population are summarized by sex and age group in Tables 1 and 2, respectively.

**Table 1:** Characteristics of participants studied

	Men	Women
Participation; %	63.7	69.3
Colonoscopy compliance; %	95.0	94.2
Total number of participants^a^	10,982	7291
Colorectal cancer (CRC)	693	339
Age (years); mean (SD)	61.4 (5.1)	60.2 (5.6)
μg Hb/g faeces; median (IQR)	219.0 (74.2–694.5))	175.3 (63.8–440.8)
Location Location (proximal side/distal side/rectum)^b^; %	18.2/70.1/11.7	21.8/64.2/14.0
Stage (I-II/III-IV/missing); %	68.0/27.6/4.4	63.7/30.8/5.5
Size (cm); mean (SD)	2.7 (1.5)	2.8 (1.6)
Advanced adenomas (AA)^c^	5188	2028
Age (years); mean (SD)	60.1 (5.4)	59.8 (5.6)
μg Hb/g faeces; median (IQR)	79.2 (35.2–229.6)	71.6 (33.2–188.6)
Location Location (proximal side/distal side/rectum)^b^; %	20.1/67.4/12.5	20.1/63.7/16.2
Number polyps; median (IQR)	3.0 (2.0–5.0)	2.0 (1.0–4.0)
Higher size polyps (mm); median (IQR)	12.0 (9.0)	12.0 (8.0)
Size of AA >9 mm; %	65.1	65.1
Size of AA >19 mm; %	13.6	12.8
AA with villous component; %	36.2	36.3
AA with severe/high-grade dysplasia; %	8.6	8.7

*SD* Standard deviation, *IQR* Interquartile range

^a^Positives

^b^Right side includes regions up to and including the splenic flexure; left side includes descending colon and up to rectum

^c^Advanced adenomas: adenomas ≥10 mm, adenoma with a villous component (i.e., tubulovillous or villous adenoma) or adenomas with severe/high-grade dysplasia

**Table 2:** Characteristics of participants stratified by sex and age

	Men < 55 years	Men 55–60 years	Men 60–65 years	Men > 65 years	Women < 55 years	Women 55–60 years	Women 60–65 years	Women > 65 years
Participation; %	59.0	63.7	67.9	66.8	65.5	70.6	72.1	69.0
Colonoscopy compliance; %	93.7	94.0	94.8	97.4	94.2	94.8	94.1	93.8
Total number of participants^a^	2415	2671	3238	2658	1775	1707	2009	1800
Colorectal cancer (CRC)	84	155	216	238	72	82	87	98
μg Hb/g faeces; median (IQR)	179.4 (56.8–536.2)	230.8 (69.6–770.4)	209.8 (70.2–682.5)	231.2 (82.4–698.7)	191.8 (77.9–542.9)	172.8 (67.6–405.3)	158 (63.2–490.4)	172.8 (54.38–443.7)
Location (proximal side/distal side/rectum)^b^; %	19.8/69.7/10.5	18.3/64.7/17.0	17.4/79.5/3.1	17.3/66.4/16.3	20.8/65.4/13.8	22.6/66.7/10.7	20.9/64.3/15.1	23.2/60.6/16.2
Stage (I-II/III-IV/missing); %	75.0/20.2/4.8	66.9/27.3/5.8	63.7/34.0/2.3	70.0/27.0/3.0	69.0/22.7/8.3	54.9/41.5/3.6	60.5/33.7/5.8	71.4/22.4/6.2
Size (cm); Mean (SD)	2.5 ± 1.6	2.5 ± 1.6	2.7 ± 1.4	2.9 ± 1.5	2.9 ± 1.5	3.0 ± 1.6	2.6 ± 1.8	2.9 ± 1.7
Advanced adenomas (AA)^c^	976	1250	1614	1348	461	498	553	516
μg Hb/g faeces; median (IQR)	78.8 (34.8–223.0)	78.8 (34.8–221.9)	84.4 (35.4–248.4)	75.8 (35.6–221.8)	71.8 (33.4–183.4)	70.2 (34.2–190.2)	71.2 (30.0–186.2)	75.6 (35.7–193.3)
Location (proximal side/distal side/rectum)^b^; %	19.4/66.2/14.4	24.3/65.1/10.6	17.3/69.4/13.3	19.3/68.9/11.8	23.5/57.8/18.7	18.4/66.5/15.1	19.1/69.2/11.7	19.5/61.2/19.3
Number polyps; median (IQR)	3.0 (1–0-4.0)	3.0 (2.0–5.0)	3.0 (2.0–5.0)	3.0 (2.0–5.0)	2.0 (1.0–3.0)	2.0 (1.0–3.0)	2.0 (1.0–4.0)	2.0 (1.0–4.0)
Higher size polyps (mm); median (IQR)	13.4 (7.5)	13.7 (7.9)	14.2 (8.5)	13.8 (10.4)	12.0 (9.7)	13.4 (8.6)	13.4 (8.4)	12.9 (7.2)
Size of AA >9 mm; %	62.4	65.2	65.3	66.4	63.4	64.2	65.2	66.1
Size of AA >19 mm; %	9.8	10.4	12.3	13.9	10.4	11.9	12.7	14.1
AA with villous component; %	35.4	36.2	35.4	37.0	34.2	36.3	35.2	36.8
AA with severe/high-grade dysplasia; %	8.0	7.8	8.5	9.0	7.4	7.8	8.2	8.9

*SD* Standard deviation, *IQR* Interquartile range

^a^Positives

^b^Proximal side includes regions from cecum up to and including the transverse colon; distal side includes splenic flexure, descending colon and sigmoid colon

^c^Advanced adenomas: adenomas ≥10 mm, adenoma with a villous component (i.e., tubulovillous or villous adenoma) or adenomas with severe/high-grade dysplasia

The proportion of false negative results was 7.6% (95% CI: 6.5–8.8). We identified 136 interval cancers (IC) and, in Table 3, the difference in characteristics of IC and screen-detected cancers (SD-C) are summarized divided into two groups, those cancers detected in participants attending for the first time (prevalent screening cancers) and those attending in subsequent rounds (incidence screening cancers).

**Table 3:** Characteristics of interval cancers and screen-detected colorectal cancer

Total	Interval cancers^a^	Screen-detected		*p*-value
		First round	Second round	
	136 (83.3%; 1st round/16.2%; 2nd round)	889	143	-
Sex				
Men; n (%)	89 (65.4)	594 (66.8)	99 (69.2)	0.79
Women; n (%)	47 (34.6)	295 (33.2)	44 (30.8)	
Age (years)				
50–54; n (%)	26 (19.1)	137 (15.4)	19 (13.3)	0.06
55–59; n (%)	32 (23.5)	195 (21.9)	42 (29.4)	
60–64; n (%)	45 (33.1)	260 (29.2)	43 (30.1)	
65–69; n (%)	33 (24.3)	297 (33.4)	39 (27.3)	
μg Hb/g faeces; median (IQR)	2.9 (0.4–11.6)^b^	201.8 (74.4–589.8)^c^	638.3 (56.8–617.2)^c^	-
Location (proximal side/distal side/rectum)^c^; %	34.3 / 33.6 / 32.1	18.1 / 67.0 / 14.9	21.6 / 66.3 / 12.1	<0.001
Stage (I-II/III-IV); %	44.8 / 55.2	66.7 / 28.4	65.7 / 24.6	<0.001
Size (cm); median (IQR)	8 (6.0–12.0)	2.5 (1.5–4.0)	2.5 (1.5–3.5)	<0.001
Time to diagnosis				
Within 1 year; n (%)	64 (47.1)			-
1–2 years; n (%)	72 (52.9)			

^a^Interval cancers after a negative test result in the previous round

^b^Median μg Hb/g faeces at time of negative screening test result. **Median μ Hb/g faeces at time of positive screening test result

^c^Proximal side includes regions from cecum up to and including the transverse colon; distal side includes splenic flexure, descending colon and sigmoid colon

### Programme performance indicators and test characteristics

The positive predictive values (PPV) for AN, both for the study group and in each sex and age stratified groups of participants, are shown in Tables 4 and 5. Significant differences were observed at a f-Hb cut-off of 20 μg Hb/g faeces, and this patternwas maintained throughout the different f-Hb cut-offs analysed by sex. The PPV was significantly higher in men at all f-Hb cut-offs. There were also significant differences between age-specific groups in men and women, with the PPV being higher in the older population for both sexes.

**Table 4:** Test characteristics at different faecal haemoglobin concentration cut-offs by sex

	Positive predictive value^a^ (%)			Positivity rate (%)			Colorectal cancer detection rate (‰)			Advanced neoplasia detection rate (‰)		
Cut-off μg Hb/g faeces	Men	Women	*p* value	Men	Women	*p* value	Men	Women	*p* value	Men	Women	*p* value
20	52.8 (51.9–53.7)	32.9 (31.9–34.0)	< 0.0001	8.3 (8.1–8.4)	4.8 (4.7–4.9)	< 0.0001	5.2 (4.8–5.6)	2.2 (2.0–2.4)	< 0.0001	44.0 (42.9–45.1)	15.9 (15.2–16.5)	< 0.0001
25	56.0 (55.0–57.0)	36.5 (35.2–37.7)	< 0.0001	6.9 (6.8–7.1)	3.8 (3.7–3.9)	< 0.0001	4.9 (4.5–5.3)	2.1 (1.9–2.3)	< 0.0001	39.0 (38.0–40.1)	13.9 (13.3–14.5)	< 0.0001
30	57.4 (56.3–58.4)	38.3 (37.0–39.6)	< 0.0001	6.2 (6.1–6.4)	3.3 (3.2–3.4)	< 0.0001	4.7 (4.3–5.1)	2.0 (1.8–2.2)	< 0.0001	36.0 (35.0–37.0)	12.8 (12.3–13.4)	< 0.0001
35	58.6 (57.5–59.7)	39.2 (37.8–40.5)	< 0.0001	5.7 (5.6–5.8)	3.0 (2.9–3.1)	< 0.0001	4.6 (4.3–5.0)	1.9 (1.7–2.2)	< 0.0001	33.8 (32.8–34.7)	11.9 (11.3–12.4)	< 0.0001
40	59.8 (58.7–61.0)	40.6 (39.1–42.0)	< 0.0001	5.3 (5.2–5.4)	2.7 (2.6–2.8)	< 0.0001	4.5 (4.1–4.8)	1.9 (1.6–2.1)	< 0.0001	31.9 (31.0–32.8)	11.2 (10.7–11.7)	< 0.0001
50	61.5 (60.3–62.6)	42.2 (40.6–43.7)	< 0.0001	4.6 (4.5–4.7)	2.4 (2.3–2.5)	< 0.0001	4.3 (3.9–4.6)	1.7 (1.5–2.0)	< 0.0001	28.7 (27.8–29.6)	10.2 (9.7–10.7)	< 0.0001
60	62.7 (61.5–64.0)	43.3 (41.7–45.0)	< 0.0001	4.2 (4.1–4.3)	2.1 (2.0–2.2)	< 0.0001	4.1 (3.8–4.4)	1.7 (1.5–1.9)	< 0.0001	26.5 (25.7–27.4)	9.3 (8.8–9.8)	< 0.0001

^a^PPV applies for AN: defined as advanced adenoma (AA) plus colorectal cancer (CRC). Advanced adenomas: adenomas ≥10 mm, ≥3 adenoma, adenoma with a villous component (i.e., tubulovillous or villous adenoma) or adenomas with severe/high-grade dysplasia

**Table 5:** Test characteristics at different faecal haemoglobin concentration by sex and age group

	Positive predictive value^a^ [%(95% CI)]											
Cut-off μg Hb/g faeces	Women <55 years	Men <55 years	*p* value	Women 55–60 years	Men 55–60 years	*p* value	Women 60–65 years	Men 60–65 years	*p* value	Women >65 years	Men >65 years	*p* value
20	30.6 (28.5–32.7)	44.0 (42.0–45.9)	< 0.0001	34.2 (32.0–36.4)	51.9 (50.1–53.8)	< 0.0001	32.0 (30.0–34.0)	56.4 (54.7–58.0)	< 0.0001	35.2 (33.0–37.3)	57.4 (55.6–59.2)	< 0.0001
25	34.5 (32.1–36.9)	47.7 (45.5–49.9)	< 0.0001	38.2 (35.6–40.7)	55.2 (50.1–53.8)	< 0.0001	34.6 (32.3–36.9)	59.5 (57.7–61.3)	< 0.0001	38.9 (36.5–41.4)	60.2 (58.2–62.1)	< 0.0001
30	35.7 (33.1–38.3)	49.1 (46.8–51.4)	< 0.0001	40.8 (38.1–43.6)	55.2 (53.1–57.2)	< 0.0001	36.3 (33.8–38.8)	60.4 (58.5–62.3)	< 0.0001	40.2 (37.6–42.8)	61.7 (59.6–63.7)	< 0.0001
35	36.4 (33.7–39.1)	49.9 (47.5–52.2)	< 0.0001	41.6 (38.8–44.5)	56.4 (54.3–58.5)	< 0.0001	37.8 (35.1–40.4)	52.8 (50.8–54.8)	< 0.0001	41.3 (38.5–44.0)	63.3 (61.2–65.4)	< 0.0001
40	38.0 (35.1–40.9)	50.9 (48.4–53.4)	< 0.0001	42.9 (39.8–45.9)	58.0 (55.8–60.2)	< 0.0001	38.8 (36.1–41.6)	62.7 (60.7–64.7)	< 0.0001	42.8 (40.0–45.7)	64.3 (62.1–66.5)	< 0.0001
50	39.9 (36.8–43.0)	52.5 (49.9–55.2)	< 0.0001	43.6 (40.4–46.8)	60.9 (58.5–63.4)	< 0.0001	42.0 (39.0–45.0)	64.2 (62.1–66.3)	< 0.0001	44.4 (41.3–47.5)	66.0 (63.7–68.3)	< 0.0001
60	40.8 (37.5–44.2)	54.2 (51.4–57.0)	< 0.0001	45.1 (41.7–48.6)	62.3 (59.8–64.9)	< 0.0001	42.3 (39.1–45.5)	65.6 (63.4–67.8)	< 0.0001	45.4 (42.1–48.7)	67.3 (64.9–69.7)	< 0.0001
Positivity rate [%(95% CI)]												
20	3.9 (3.8–4.1)	6.1 (5.9–6.3)	< 0.0001	4.4 (4.2–4.6)	7.9 (7.7–8.2)	< 0.0001	5.3 (5.1–5.5)	9.5 (9.2–9.8)	< 0.0001	6.2 (6.0–6.5)	10.7 (10.3–11.0)	< 0.0001
25	3.1 (3.0–3.3)	5.0 (4.8–5.2)	< 0.0001	3.4 (3.3–3.6)	6.6 (6.4–6.9)	< 0.0001	4.1 (4.0–4.3)	8.1 (7.8–8.3)	< 0.0001	5.0 (4.8–5.3)	9.0 (8.7–9.4)	< 0.0001
30	2.8 (2.6–2.9)	4.5 (4.3–4.7)	< 0.0001	3.0 (2.8–3.2)	6.0 (5.7–6.2)	< 0.0001	3.6 (3.0–3.4)	7.3 (7.0–7.5)	< 0.0001	4.5 (4.3–4.7)	8.1 (7.8–8.5)	< 0.0001
35	2.5 (2.4–2.6)	4.1 (3.9–4.3)	< 0.0001	2.7 (2.6–2.9)	5.4 (5.2–5.7)	< 0.0001	3.2 (3.0–3.4)	6.7 (6.5–7.0)	< 0.0001	4.1 (3.8–4.3)	7.5 (7.2–7.8)	< 0.0001
40	2.3 (2.1–2.4)	3.7 (3.6–3.9)	< 0.0001	2.5 (2.3–2.6)	5.0 (4.8–5.3)	< 0.0001	2.9 (2.8–3.1)	6.2 (6.0–6.5)	< 0.0001	3.7 (3.5–4.0)	6.9 (6.6–7.2)	< 0.0001
50	2.0 (1.9–2.1)	3.3 (3.1–3.4)	< 0.0001	2.2 (2.1–2.4)	4.4 (4.2–4.6)	< 0.0001	2.5 (2.4–2.7)	5.5 (5.2–5.7)	< 0.0001	3.3 (3.1–3.5)	6.1 (5.8–6.4)	< 0.0001
60	1.8 (1.6–1.9)	2.9 (2.8–3.1)	< 0.0001	2.0 (1.8–2.1)	3.9 (3.7–4.1)	< 0.0001	2.3 (2.1–2.4)	5.0 (4.8–5.2)	< 0.0001	2.8 (2.7–3.0)	5.5 (5.2–5.8)	< 0.0001
Colorectal Cancer (CRC) Detection Rate [‰ (95% CI)]												
20	1.6 (1.2–2.0)	2.2 (1.7–2.6)	0.053	2.1 (1.7–2.6)	4.5 (3.8–5.2)	< 0.0001	2.3 (1.8–2.7)	6.4 (5.6–7.3)	< 0.0001	3.5 (2.8–4.1)	9.3 (8.1–10.4)	< 0.0001
25	1.5 (1.2–1.9)	2.0 (1.6–2.4)	0.107	2.0 (1.6–2.4)	4.3 (3.6–5.0)	< 0.0001	2.1 (1.7–2.6)	6.1 (5.3–6.9)	< 0.0001	3.2 (2.6–3.9)	8.7 (7.6–9.8)	< 0.0001
30	1.5 (1.1–1.8)	1.9 (1.5–2.3)	0.127	2.0 (1.5–2.4)	4.1 (3.4–4.8)	< 0.0001	2.1 (1.6–2.5)	5.9 (5.1–6.7)	< 0.0001	3.0 (2.4–3.6)	8.4 (7.3–9.5)	< 0.0001
35	1.5 (1.1–1.8)	1.9 (1.5–2.3)	0.127	1.9 (1.5–2.3)	4.0 (3.3–4.7)	< 0.0001	2.0 (1.6–2.5)	5.8 (5.0–6.6)	< 0.0001	2.9 (2.3–3.5)	8.3 (7.2–9.4)	< 0.0001
40	1.4 (1.1–1.8)	1.8 (1.5–2.3)	0.150	1.8 (1.4–2.2)	3.9 (3.3–4.6)	< 0.0001	2.0 (1.5–2.4)	5.5 (4.8–6.3)	< 0.0001	2.8 (2.2–3.4)	8.0 (7.0–9.1)	< 0.0001
50	1.4 (1.0–1.7)	1.7 (1.3–2.1)	0.241	1.7 (1.3–2.1)	3.7 (3.1–4.4)	< 0.0001	1.8 (1.4–2.2)	5.3 (4.5–6.0)	< 0.0001	2.6 (2.0–3.2)	7.8 (6.8–8.9)	< 0.0001
60	1.3 (1.0–1.6)	1.6 (1.2–1.9)	0.328	1.7 (1.3–2.0)	3.6 (3.0–4.2)	< 0.0001	1.7 (1.3–2.1)	5.0 (4.3–5.8)	< 0.0001	2.5 (2.0–3.1)	7.6 (6.5–8.6)	< 0.0001
Advanced Neoplasia (AN) Detection Rate [‰ (95% CI)]												
20	12.1 (11.1–13.0)	26.9 (15.3–28.5)	< 0.0001	15.0 (13.8–16.2)	41.3 (39.2–43.4)	< 0.0001	16.9 (15.6–18.2)	53.6 (51.2–55.9)	< 0.0001	21.9 (20.3–23.6)	61.2 (58.3–64.1)	< 0.0001
25	10.8 (9.9–11.8)	23.9 (22.5–25.4)	< 0.0001	13.1 (12.0–14.2)	36.5 (34.6–38.5)	< 0.0001	14.4 (13.2–15.5)	47.9 (45.7–50.2)	< 0.0001	19.6 (18.0–21.2)	54.2 (51.5–56.9)	< 0.0001
30	9.9 (9.0–10.7)	21.9 (20.5–23.3)	< 0.0001	12.3 (11.2–13.3)	33.6 (31.7–35.4)	< 0.0001	13.0 (11.9–14.1)	44.0 (41.9–46.1)	< 0.0001	18.0 (16.5–19.5)	50.1 (47.5–52.8)	< 0.0001
35	9.1 (8.2–9.9)	20.3 (19.0–21.7)	< 0.0001	11.4 (10.4–12.4)	31.5 (29.6–33.3)	< 0.0001	12.1 (11.1–13.2)	35.6 (33.7–37.5)	< 0.0001	16.8 (15.4–18.2)	47.6 (45.0–50.1)	< 0.0001
40	8.6 (7.8–9.4)	19.1 (17.8–20.4)	< 0.0001	10.7 (9.7–11.7)	30.0 (28.2–31.8)	< 0.0001	11.4 (10.4–12.4)	39.0 (37.0–47.1)	< 0.0001	16.0 (14.6–17.5)	44.6 (42.1–47.1)	< 0.0001
50	7.9 (7.1–8.7)	17.2 (15.9–18.4)	< 0.0001	9.7 (8.8–10.7)	26.7 (25.1–28.4)	< 0.0001	10.7 (9.7–11.7)	35.2 (33.3–37.1)	< 0.0001	14.5 (13.1–15.8)	40.4 (38.1–42.8)	< 0.0001
60	7.2 (6.5–8.0)	16.0 (14.8–17.2)	< 0.0001	8.9 (8.0–9.8)	24.5 (22.9–26.1)	< 0.0001	9.7 (8.7–10.7)	32.8 (30.9–34.7)	< 0.0001	12.9 (11.7–14.2)	37.1 (34.9–39.4)	< 0.0001

^a^PPV applies for AN: defined as advanced adenoma (AA) plus colorectal cancer (CRC). Advanced adenomas: adenomas ≥10 mm, ≥3 adenoma, adenoma with a villous component (i.e., tubulovillous or villous adenoma) or adenomas with severe/high-grade dysplasia

The positivity rate for the range of f-Hb cut-offs assessed was also higher in men and the difference with women was also significant, with the positivity decreasing with increasing f-Hb cut-off. The positivity was lower for all age groups in both sexes as the f-Hb cut-off increased, being higher in older men and women, and with significant differences by sex (Tables 4 and 5).

The CRC detection rate (CDR) was higher in men than in women and in older subjects, with significant differences for all f-Hb cut-offs (Tables 4 and 5). In men, the CDR decreased from 5.2‰ (95% CI: 4.8–5.6) to 4.1‰ (95% CI: 3.8–4.4) and in women from 2.2‰ (95% CI: 2.0–2.4) to 1.7‰ (95% CI: 1.5–1.9). The advanced neoplasia detection rate (ANDR) was also higher in men at a f-Hb cut-off of 20 μg Hb/g faeces (44.0‰ [95% CI: 42.9–45.1]), with a significant difference with respect to women, for whom the ANDR was lower (15.9‰ [95% CI: 15.2–16.5]). This significant difference was also maintained at different f-Hb cut-offs. The ANDR was higher in older groups in both sexes, with significant differences by sex for all f-Hb cut-offs (Tables 4 and 5). In any case, the ANDR in men over 60 years remained higher than that of women.

### Colonoscopy savings and the risk of losses in the detection of advanced colorectal Neoplasia

**Fig. 2:**
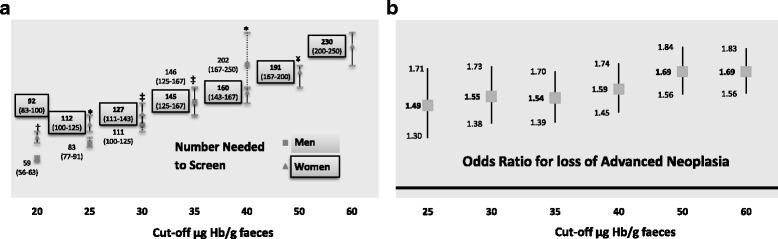
Number Needed to Screen to detect Advanced Neoplasia (AN) (**a**) and the Odds Ratio for the loss in detection of AN (**b**) Men versus women through increasing the faecal haemoglobin cut-off. (*p < 0.001; ^†^ *p* < 0.05; ^‡^no significance). (^¥^Cut-off 50 μg Hb/g faeces in men = 509 [95% CI: 333–1000])

A lower NNS to detect one AN (59; 95% CI: 56–63) was seen in men at a f-Hb cut-off 20 μg Hb/g faeces compared to 92 (95% CI: 83–100) for women. On increasing the f-Hb cut-off, NNS increased to 230 for women at a f-Hb cut-off of 60 μg Hb/g faeces. The differences between men and women were significant at f-Hb cut-offs of 20 and 25 μg Hb/g faeces but not at higher cut-offs (30 and 35 μg Hb/g faeces), as shown in Fig. 2a.

A logistic regression analysis was performed to determine the risk of loss in the detection of AN by increasing the f-Hb cut-off (Fig. 2b). The risk is higher in men than in women and this risk increases significantly upon increasing the f-Hb cut-off from 1.49 (95% CI: 1.30–1.71) to 1.69 (95% CI: 1.56–1.83).

**Fig. 3:**
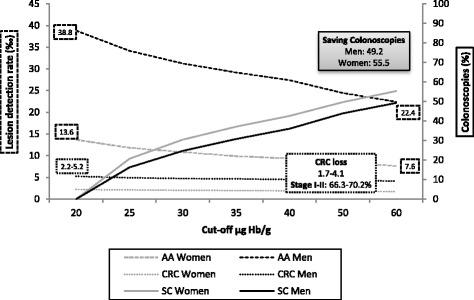
Relation between saving colonoscopies (SC) and lesion loss upon increasing the faecal haemoglobin concentration cut-off by sex. Dotted lines represent lesion detection rates (for colorectal cancer (CRC) and advanced adenoma (AA)) and solid lines saved colonoscopies. The left Y axis represents lesion detection rate and the right Y axis the percentage of colonoscopies saved. Saving Colonoscopies: the percentage of colonoscopies that will not be performed in the programme by increasing the f-Hb cut-off, due to the reduction of positivity rate

The colonoscopy saved by increasing the f-Hb cut-off in the case of women increases to 55.5% (*N* = 4273). As such, the savings made in terms of colonoscopies are offset by the loss in detection of CRC and AA (Fig. 3). The loss of AA in women can be as high as 43.3% (*N* = 962), and 22.9% for CRC (*N* = 81). Around 19.1% of the colonoscopies saved upon increasing the f-Hb cut-off to 25 μg Hb/g faeces will have an AN, and this percentage rises to 24.4% on increasing the f-Hb cut-off to 60 μg Hb/g faeces It can also be seen that the CRC missed were diagnosed mostly at an early stage (Stage I-II: from 70.2% in men to 66.3% in women).

**Fig. 4:**
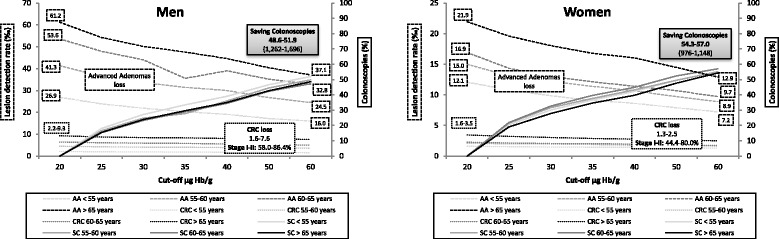
Relation between saving colonoscopies (SC) and lesion losses upon increasing the cut-off level of the FIT by sex and age group. Dotted lines express lesion detection rates (colorectal cancer (CRC) and advanced adenoma (AA)) and solid lines saved colonoscopies. The left Y-axis represents lesion detection rate and the right axis the percentage of colonoscopies saved. Saving Colonoscopies: the percentage of colonoscopies that will not be performed in the programme by increasing the f-Hb cut-off, due to the reduction of positive rate

Colonoscopy savings increased in all age groups on increasing the f-Hb cut-off in both sexes. However, as can be seen from Fig. 4, there is no substantial difference in this saving by age group (from 48.6 to 51.9% in men and 54.3 to 57.0% in women). However, an analysis of the decrease in CRDR and ANDR showed a considerable difference between age groups in both sexes. Thus, in men, the AADR decreased by 24.1 and 10.9‰, in the oldest group and in the youngest groups respectively, whereas in women it decreased by 9.0‰ in the oldest group and by 4.9‰ in the youngest. A similar pattern was observed in CDR and, depending on the age group analysed, the diagnoses of early-stage CRC not detected could be as high as 86.4% in men and 80.0% in women.

## Discussion

We have compared CRC screening with FIT at different f-Hb cut-offs in a large population aged between 50 and 69 years. To our knowledge, there have been few previous studies of sex and age related differences in population-based FIT screening programs.

In our study, a total of 444,582 persons were invited to participate in the Basque Country CRC Screening Programme. This large number of participants facilitated the performance of a reliable and robust statistical analysis to determine whether a simple, single f-Hb cut-off should be used for different populations without increasing the interval cancer rate, thus allowing the provision of insight for others running similar programmes.

CRC screening programmers in a number of countries have encountered higher than expected positivity [36], thus leading to overwhelming demand for scarce colonoscopy resources and a need to increase the f-Hb cut-off to lower the number of referrals. In consequence, data on the performance of FIT in men and women are of key importance due to the current widespread and growing use of FIT in population-based CRC screening programmes.

We observed a higher PPV for AN and higher detection rates for CRC and AN than other programmes, these results could be due to the high rate of compliance to colonoscopy assessment, that allowed a minimal loss of neoplasm detection As reported in recently published studies [26, 37], higher positivity was found in men at the full range of f-Hb cut-offs. This pattern is also consistent when comparing older men and women against younger ones, with these variables being higher in older groups. A decision on whether to adjust the age at which screening begins also requires taking into consideration whether the recommended age for men should be younger or the recommended age for women older. In this regard, Sung et al. [38], in the Asia Pacific consensus recommendations for CRC screening, suggested that women may start screening at later ages due to the relatively low incidence of CRC at 50–55 years. Similarly, Brenner suggested that the optimal age for screening initiation should be five years younger for men than for women. Despite this, European guidelines recommend that screening programs for CRC should start at age 50 years for both men and women of average risk [10]. However, the question of using different f-Hb cut-offs for men and women and/or younger and older participants remains unsolved. Differences in the epidemiological pattern of CRC among sexes have been identified during the last years [39]. Hence, it is a matter of discussion if the screening must be implemented on the basis of same sex, age and f-Hb cut-off.

Recent studies [22, 27] have concluded that FIT has a higher sensitivity and a lower specificity for CRC in men than in women and therefore that equal test characteristics can be achieved by allowing different f-Hb cut-offs for the sexes. However, Kapidzic et al. [26], observed that there were no significant differences between men and women in PPV at a f-Hb cut-off of 10 μg Hb/g faeces, thus meaning that the chance that a colonoscopy is unnecessary after a positive test result is the same. It was suggested that, if the same differences were to persist between men and women in a larger sample, the differences in PPV would become significant, and this is exactly what we have observed in our study, in which the differences between men and women have remained statistically significant. However, can we therefore argue that it would be better to increase the f-Hb cut-off for women? According to the results of Kapidzic et al. [26], the PPV could be improved using a higher f-Hb cut-off in women; however, this would be at the expense of increasing the NNS as this increases at higher f-Hb cut-offs.

It may take approximately 10 years from the appearance of the first lesion with abnormal histopathology to develop a possible malignant lesion. In 2007, Brenner et al. [39] showed that the risk of transition from AA to CRC was similar for men and women, but increased with age. Some studies [40, 41] have reported significantly higher detection rates for AN and CRC with colonoscopy for men than for women in all age groups, thus suggesting that male sex constitutes an independent risk factor for colorectal neoplasia. Such studies recommended sex-specific ages for screening. These differences are similar to those observed in our study.

Colonoscopy resource can be key to defining the strategies and characteristics adopted in screening programmes. Indeed, the additional number of colonoscopies that need to be performed may become an important factor when deciding whether to establish any such programme. We observed that the saving in colonoscopies increased consistently in both sexes and in all age groups as the f-Hb cut-off was increased. It might seem appropriate to increase the f-Hb cut-off since this would a lower the number of colonoscopies required. However, when increasing the f-Hb cut-off, the risk of lowering the ANDR increases significantly in both sexes and in all age groups. The proportion of IC could be higher in men than in women and in older groups. Thus, an increase in the f-Hb cut-off could increase the loss from 7.9 to 28.1% in men and from 5.1 to 22.9% CRC in women. This loss in the detection of CRC is consistent over all age groups. Moreover, taking into account that most of those with CRC would be diagnosed in their early stages, this would go against the principles of preventive screening programmes. These results are consistent with those published recently by Digby et al. [42], who concluded that CRC screening programmes would benefit from using low f-Hb cut-off to gain lower IC proportions as well as higher sensitivity and detection of earlier stage disease, but at the cost of increased demand for colonoscopy.

Recent studies suggested the potential benefits of using a risk prediction model including f-Hb in CRC screening [18, 29, 31, 43] to improve the effectiveness of screening strategies. Future studies performed should therefore be designed to evaluate the benefits of implementing models according to the different risks of different groups according to sex and age. Some studies have suggested that other factors could be used to determine the optimal cut-off values for men and women, and that the combination of these data with microsimulation models could improve the implementation of screening programmes [28, 44].

One of the main strengths of the current study was the large number of participants evaluated, all of whom were recruited in an organized, population-based screening programme, coordinated and systematically evaluated at a single centre. The lack of studies published to date with real data from such a FIT-based programme and with a participation rate of more than 65% (the level recommended in the European guidelines [10]) is also worth noting.

However, several limitations have to be acknowledged. The study included assessment of the effects of sex and age but no other possible confounding factors, such as socio-economic status which has been shown to affect f-Hb [36, 45], though they could be retrospectively explored on the basis of a case/control nested analysis. Furthermore, Brenner [38] suggested that appropriate differentiation of age at initiation of CRC screening by sex might be equally or more relevant from a public health point of view than the widely used differentiation by family history.

## Conclusions

In conclusion, this population-based study provides relevant information on the performance of a realistic FIT-based colorectal screening programme in men and women at different f-Hb cut-offs. Men have higher PPV, CDR and ANDR, which results in a lower NNS when compared to women, and this pattern is consistent when comparing younger and older groups. However, given the assessed loss in detection of AN and CRC, most of them in their early stages, it may be that the f-Hb cut-off that is going to be implemented should not be change only by sex or age, at least initially, in accordance with the recommendations of the European guidelines, in order not to increase the ratio of interval cancers, which is another important variable to examine.

## Abbreviations

AA: Advanced adenoma
AN: Advanced colorectal neoplasia
ANDR: Advanced neoplasia detection rate
CDR: Cancer detection rate
CRC: Colorectal cancer
f-Hb: Faecal haemoglobin concentration
FIT: Faecal immunochemical test
FOBT: Faecal occult blood test
Hb: Haemoglobin
IC: Interval cancers
NNS: Number needed to screen to detect one case
PPV: Positive predictive value
SD-C: Screen-detected cancers
